# Use of Insect Promoters in Genetic Engineering to Control Mosquito-Borne Diseases

**DOI:** 10.3390/biom13010016

**Published:** 2022-12-21

**Authors:** Vanessa Bottino-Rojas, Anthony A. James

**Affiliations:** 1 Department of Microbiology and Molecular Genetics, University of California, Irvine, CA 92697, USA; 2 Department of Molecular Biology and Biochemistry, University of California, Irvine, CA 92697, USA

**Keywords:** regulatory DNA, gene expression, marker genes, mosquito transgenesis

## Abstract

Mosquito transgenesis and gene-drive technologies provide the basis for developing promising new tools for vector-borne disease prevention by either suppressing wild mosquito populations or reducing their capacity from transmitting pathogens. Many studies of the regulatory DNA and promoters of genes with robust sex-, tissue- and stage-specific expression profiles have supported the development of new tools and strategies that could bring mosquito-borne diseases under control. Although the list of regulatory elements available is significant, only a limited set of those can reliably drive spatial–temporal expression. Here, we review the advances in our ability to express beneficial and other genes in mosquitoes, and highlight the information needed for the development of new mosquito-control and anti-disease strategies.

## 1. Introduction

Mosquito-borne diseases are one of the greatest challenges to global health [1]. *Anopheles* mosquitoes are the main vectors of human malaria parasites; *Aedes* species are major transmitters of arboviruses, including dengue, chikungunya, and Zika; and *Culex* mosquitoes are prominent vectors of viruses that cause encephalitic infections, including West Nile virus, and nematode parasites that cause lymphatic filariasis. Classical disease control methods, including repellents and bed nets, target bite prevention and mosquito elimination, typically using chemical insecticides [2]. However, disease incidences remain high, and resistance to commonly used insecticides is increasingly present in wild mosquito populations [3]. In order to supplement insecticide-based control strategies, the use of genetically engineered mosquitoes has been proposed to provide next-generation tools for disease prevention, and these include genetic-based vector population elimination or a reduced pathogen transmission capacity [4,5].

Advances in the knowledge of vector–pathogen interactions and mosquito biology, combined with the development of genomic data and sophisticated tools for genetic editing, provide opportunities to improve transgenic technologies in major mosquito vectors. Transgenesis experiments are essential to investigate endogenous gene function and to introduce exogenous DNA products desired to mitigate pathogen transmission [4,6]. Current gene drive research is centered mainly on drives based on the CRISPR-Cas9 genome-editing toolset, and the proposed strategies use pre-characterized promoter and terminator elements, each driving tissue-specific transgene expression as required for different functions in the germline and in various somatic tissues. However, compared to the knowledge accumulated on transcriptional regulation in the vinegar fly, *Drosophila melanogaster*, little is known about the regulatory genome of mosquitoes. In fact, the regulatory networks of most mosquito genes remain understudied from a mechanistic perspective [7].

Computational predictions and comparative genomics tools have assisted in the identification of *cis*-acting regulatory regions and transcriptional enhancers in mosquitoes [8,9]. Additionally, the functional fragments of gene control sequences have been defined primarily through transposon-mediated transgenesis experiments [10,11,12]. Nevertheless, a comprehensive understanding of mosquito regulatory biology requires combining and cross validating data generated using direct, indirect and in silico approaches. Recent efforts are in place to further characterize mosquito gene regulatory networks in vivo and provide new insights into mechanisms controlling mosquito functional gene expression [7,13].

Here we review the various promoters that have been shown to drive transgene expression in mosquitoes and their utility for biotechnology-based control approaches, as well as the valuable insights into mosquito regulatory biology they provide.

## 2. Genetic Engineering Toolbox

The ability to manipulate gene expression in specific tissues at specific times in development is key to understanding mosquito biology and developing genetic means for vector control [14]. This work is facilitated in mosquitoes through the use of bi-partite expression systems such as the Gal4-UAS system [10,15,16]. For example, a recent contribution using this system expanded the genetic tools available to study gene function in hemocytes by characterizing the gene expression pattern driven by the *Drosophila hml* promoter in *An. gambiae* adult females [17]. Similarly, in an effort to obtain multi-tissue ubiquitous-like expression of transgenes, promoters of highly conserved ‘housekeeping’ genes such as *polyubiquitin* [18] have been investigated in mosquitoes, with successful validation of constitutive transcriptional activities [19,20]. Exogenously derived promoters and control sequences from genes such as *heat-shock protein 70*, *actin5c* and *ubiquitin* from *D. melanogaster*, and the baculovirus *immediate-early* (IE1), have also been useful in mosquito transgenesis [21,22,23,24,25,26].

Sustained, easily scored marker gene expression is desirable during screening for transgenic individuals when making new or maintaining previously established lines. This has been traditionally achieved by the use of a variety of viral and insect promoters, due to their lack of organism and tissue-specificity, to direct the expression of a fluorescent protein-encoding or other visible marker gene. The most frequently used and best characterized promoters come from *D. melanogaster* or baculoviruses and have been used successfully to express both exogenous and modified endogenous genes in mosquitoes or mosquito cell lines [12,25,26,27,28,29,30,31,32,33,34]. Later, genomic sequences derived from regions adjacent to the 5′-end (upstream) of endogenous heat shock protein-encoding genes were shown to be capable of driving marker expression in *Ae. aegypti*, both transiently in cells and embryos and through the stable integration of transgenes [35,36]. Although the most commonly used promoter for marker visualization is *3xP3* [37,38], which is remarkably visible in the optic nerves of larvae and pupae [39], identification of positive individuals can be difficult in weaker phenotypes or in later stages of development due to dark eye pigmentation [40]. In such cases, marker genes driven by strong constitutive promoters can be advantageous, particularly if there is a need to reliably identify transgenic mosquitoes in a wider range of stages [41], such as in field applications.

Initial work to define and characterize mosquito promoters in vivo relied on Class II transposable elements (transposons) to integrate modified endogenous and exogenous gene constructs stably and heritably into vector genomes. Class II elements are DNA-based and comprise a gene (and regulatory elements) encoding a transposase enzyme flanked by inverted repeat sequences of varying complexity and length (length and sequence are characteristic of each family of transposons). Complete (autonomous) elements are able to mobilize (excise and integrate) through either a conservative (no net increase in copy number) or replicative (increase in copy number) mode, thereby changing their linkage relationships in the genome [41]. Following the inability to adapt the *P* element, first discovered in *D. melanogaster*, to mosquito species, new discoveries identified a number of elements, *Hermes*, *Mos 1 mariner*, *Minos*, and ultimately, *piggyBac*, that work well in both anopheline and culicine mosquitoes [42].

More recently, high-efficiency genome engineering applications, such as those based on CRISPR-Cas9 technologies, have supplanted most applications of transposable elements. Their ability to target the integration of DNA to a preselected site in the genome can be used to control or mitigate variations of transgene performance resulting from position-site effects often encountered when using transposable elements [42]. The Cas9-based systems require a ubiquitously expressed guide RNA (gRNA) sequence to direct nuclease cleavage activity at the preselected site as a first step in integration or modification. In mosquitoes, RNA Polymerase III (Pol III) promoters have been used for genetic control strategies that depend on gRNA or RNAi [43] expression. In particular, the *U6* RNA polymerase III gene promoters are ideal for non-coding RNA expression due to their nucleus-associated transcription without the 5′- and 3′-end mRNA modification associated with Polymerase II gene expression. Endogenous *U6* regulatory sequences have been used to drive gRNA expression across different mosquito species, with varying degrees of activity [40,44,45,46]. To complement the tools used for efficient transgene transmission in mosquitoes, we discuss below the multiple sequences used to drive expression of Cas9 in the germline, a capability essential for gene drive development.

## 3. Germline-Specific Promoters

Two general genetic strategies for mosquito population management have been envisioned: population suppression, and population replacement with individuals that are refractory to disease transmission. Several gene-drive based versions of these strategies require the use of regulatory sequences that drive expression primarily or exclusively in the germline, particularly the ones that utilize engineered site-specific homing endonucleases. When expression of an endonuclease such as Cas9 is activated in the male and/or female germline in a hemizygote, cleavage of the target site on the wild-type chromosome followed by homology-directed DNA repair results in an increase in the frequency of the drive transgene in the population [47].

In mosquitoes, expression of transgenes in germline-specific patterns were achieved using notable regulatory sequences. The regulatory regions of the *β2-tubulin* gene have been utilized to drive testis-specific marker expression [48,49]. The germline-specific regulatory promoter and untranslated regions from the *vasa*, *nanos*, and *zero population growth* (*zpg*) genes have been used to direct expression of the Cas9 nuclease in male and female germ cells as components of gene-drive systems [40,50,51,52,53]. In addition, sex-specific expression of fluorescent markers can be exploited for an efficient high-throughput sex separation during mosquito rearing. This strategy has been pursued using the *β2 tubulin* promoter in *Ae. aegypti* [48] and *An. stephensi* [49], and in *An. gambiae*, where the use of the *doublesex* (*dsx*) promoter [54] permits early larval separation due to its selective expression-driven pattern in male larvae at early developmental stages [55].

Considering gene drives, different Cas9 expression constructs support the conclusion that promoter-dependent Cas9 transcript localization may play a critical role in drive integration outcomes [56,57]. It has been demonstrated that successful drive conversion in the male germline occurs without subsequent formation of resistance alleles in the embryo due to paternally deposited Cas9 [57]. In contrast, in zygotes with maternal deposition of Cas9/gRNA complexes, the physical distance between the paternal and maternal chromosomes within the embryo may prevent homology-directed repair and result in a potentially drive-resistant allele [40,58]. Furthermore, lower levels of paternally transmitted Cas9 in the embryo can minimize off-target and toxicity effects. Additionally, the *nanos* promoter significantly lowered somatic Cas9 expression compared to the *vasa* promoter, supporting the conclusion that it is a better choice in drive strategies where gene disruption in somatic cells could have fitness costs [59]. Recently, an examination of transcript distribution patterns of Cas9 transgenes driven by the *vasa* or *nanos* promoters in the germline of transgenic *Anopheles* mosquitoes showed an overall strong concordance between promoter-driven Cas9 and endogenous gene expression patterns for both drive systems in males, but also distinct colocalization patterns for the two drives in female reproductive tissues [53]. Despite the imperfect overlap of transgene vs. endogenous transcript patterns, transgenic *nanos*-Cas9 mosquitoes display highly efficient drive performance [58,60].

The *zpg* control DNA sequences have suitable performances in *An. gambiae* drive studies [61,62], potentially due to reduced Cas9 expression levels [53]. In *Ae. aegypti*, a comprehensive assessment revealed transcriptional regulatory regions able to drive female germline-specific, and female and male germline-specific expression, with overlapping but also distinct transcriptional patterns [63]. Using these, different Cas9 strains engineered achieved great mutagenic efficiency and specificity [34]. Additionally, a study of *Ae. aegypti* developmental transcriptomes [64] led to the identification a novel female germline and early zygote promoter from the transcription factor *bZip1* [65]. It was demonstrated that transgenic lines in which the *bZip1* promoter expresses a fluorescent marker protein follow the same pattern of expression as the endogenous gene, although the genomic fragment chosen appears to be strongly repressed by position effects.

Hence, the use of different germline-specific control DNA sequences has provided initial resources toward understanding the basis for differing drive properties and the identification of regulatory elements that will be instrumental in furthering our understanding of mosquito biology and control.

## 4. Population Suppression

Genetics-based population suppression can be achieved through mass-release of mosquitoes carrying dominant-lethal, sex-conversion, or female reproductive damaging transgenes. Dominant-lethal and female reproductive damaging approaches usually require spatially restricted expression of a deleterious gene product under the control of specific regulatory elements to achieve their designed outcomes. Additionally, to maintain breeding populations of transgenic strains, these lethal or damaging effector genes must be under conditional regulation so that lethality or sterility only occurs under non-permissive conditions [66]. The promoter and control DNA derived from the flight muscle-specific *Actin-4* gene in *Ae. aegypti* [67] and an antibiotic-repressible lethal factor were used to create a female-specific flightless phenotype strain [68]. This approach was also shown to work in a related species, *Ae. albopictus* [69] and was successfully adapted to *An. stephensi* [70], with the use of orthologue endogenous regulatory sequences. Moreover, while early studies in *Cx. quinquefasciatus* transgenesis proved the functional conservation of the flight muscle *D. melanogaster act88F* promoter [71], its use to drive effector transgene expression has not yet been accomplished for this species.

Some insect promoters can be exploited for developing mosquito control strategies to reduce vector populations by female-to-male sex conversion, or to aid in sterile insect techniques that require releasing only non-biting males. For example, the ectopic expression of the Y chromosome-linked signal gene *Yob* under the control of the germline promoter *vasa* generated a partial female lethal phenotype in *An. gambiae* [72]. However, complete penetrance of the lethal phenotype may require the use of promoters that are more active during the early zygotic stage. This has been achieved in *An. stephensi*, where a male-only phenotype was achieved by expression of an autosomally integrated construct consisting of the male-determining gene, *Guy1*, driven by its own endogenous promoter [73]. Additionally, successful conversions of females into fertile males with all male-specific sexually dimorphic features were achieved using the native promoter of the male-determining factor *Nix* in *Ae. aegypti* and *Ae. albopictus* [74,75].

A newly developed molecular tool expanded the flexibility of suppression technologies by engineering a paralysis-inducing neurotoxic synthetic effector designed to be secreted by the adult fat body following a blood-meal, under the control of the *vitellogenin* (*Vg*) promoter in *Ae. aegypti* [76]. This makes it possible to dissociate the temporal and spatial expression patterns of an effector, and allow the use of a wider panel of endogenous regulatory components for building genetic lethal systems.

## 5. Population Modification

**Figure 1:**
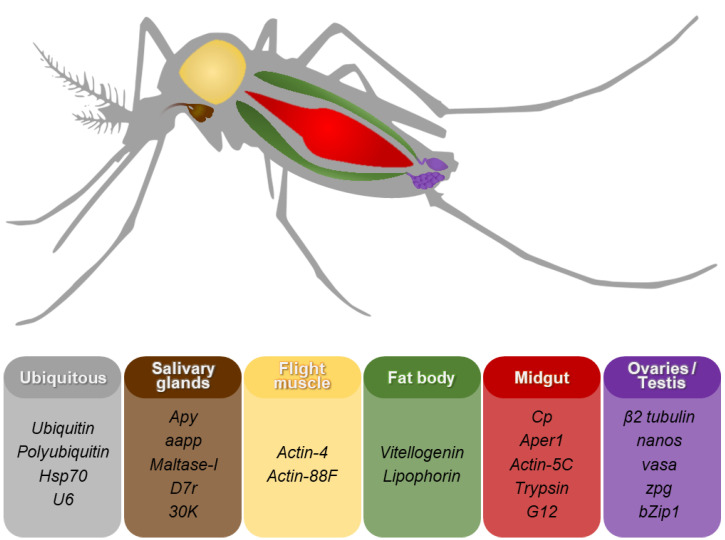
Genes whose promoters and 5′- and 3′ end DNA sequences are used in genetic engineering of mosquitoes. The promoters and control DNA of a number of genes have been used to drive expression of genetic components or effector molecules in different tissues of the mosquito. Details and references on the promoters listed are included in the text. Abbreviations: *aapp*, anopheline antiplatelet gene; *Aper1*, adult peritrophic matrix gene; *Apy*, apyrase; *Cp*, zinc carboxypeptidase A1; *D7r*, D7-related gene; *Hsp70*, heat-shock protein 70.

Mosquito genomic studies have long focused on the design of engineered genes under the control of promoter-regulatory DNA to drive site-specific expression in infection-relevant tissues (‘compartments’ [77]). In addition to spatial considerations, the time of transgene-mediated protein synthesis relative to pathogen arrival in each of these compartments was considered. This is a favorable design feature in engineering mosquitoes to minimize potential transgenesis-related fitness costs by restricting the expression of transgenes to the infection-relevant sex, developmental stage, and mosquito body compartments in which the pathogens are found. Many endogenous promoters have been used to drive transgene expression in mosquitoes and examples of these are listed in Figure 1.

As the midgut is the first tissue encountered by newly introduced parasites and arboviruses, the regulatory DNA of midgut-specific genes, particularly those that are expressed at high levels in response to a blood-meal, are ideal candidates for directing the expression of effector genes in this compartment. The most widely characterized regulatory regions are from digestive enzyme-encoding genes such as *trypsin* [78,79,80,81] and *carboxypeptidase* (*Cp*) [82]. The control DNA sequences of other genes can be useful for strict female-specificity, such as the *An. gambiae G12* gene [81], or being abundantly expressed in the midgut even prior to a blood meal, such as *peritrophin* (*Aper1*) and *actin5C* [12,83]. A number of robust anti-pathogen strategies have been developed using *Cp* gene ortholog promoters in *Anopheles* species [84,85] and *Aedes aegypti* [86]. However, the use of this promoter also has been associated with lowered fitness of transgenic mosquitoes, linked to the action of the transgenes themselves. Mosquitoes with *Cp*-driven *Akt* signaling have an impacted lifespan, likely due to leaky expression at the non-blood-fed stage [87], and certain exogenous antimicrobial peptides can exert internal damage to the midgut or cause undesired physiological effects [85,88].

During subsequent stages of infection, the pathogens traverse the midgut wall (intracellularly and/or extracellularly) and migrate through the open circulatory system (hemocoel) to the mosquito salivary glands (*Plasmodium* parasites and arboviruses) or proboscis (filarial nematodes). Hence, effector gene expression targeted to the mosquito hemocoel can impact pathogen migration. The *Vg* gene *cis*-acting DNA sequences are the most widely used [23,26,89,90,91] to induce late-digestion and sex-specific expression of desired gene products in the fat body for secretion into the hemolymph. This gene has a restricted temporal profile of expression that peaks around 24 h after a blood meal and returns to basal level by 48 h. However, for sustained *Vg*-driven expression, the promoter can be re-activated by additional blood meal(s) [92]. Similar to *Cp*-induced expression, different combinations of molecules and *Vg* expression systems have contrasting impacts on mosquito survival and consequent transgene integration into populations. For example, expression of the peptide SM1 driven by the *Vg* promoter imposes a significant fitness load to transgenic mosquitoes [93], but the same does not occur on individuals with the *Cp* control DNA driving expression of SM1 [88]. Nonetheless, a number of highly effective transgenic lines that target multiple infection stages through multi-effector expression using both *Cp* and *Vg* do not show impaired life spans [85,92]. Additionally, heterologous [94] or mosquito promoter regions can be used to drive salivary gland-specific transgene expression, including those from *Maltase-I*, *D7r* and *apyrase* [21,95,96,97], and the *anopheline antiplatelet* gene (*aapp*) [98,99,100]. Transgene products were expressed in *Ae. aegypti* under the control of a functional bi-directional *30K* gene promoter, significantly reducing Dengue virus titers in mosquito salivary glands [101]. The promoter region of *aapp* also has been used to induce production, secretion, and host inoculation of a malarial protein through *An. stephensi* saliva [102].

Finally, mosquito promoters of immune-modulated genes are potentially useful for being sensitive to pathogen presence in the system [103]. In addition, the importance of *cis*-acting mutations on detoxification enzyme genes for insecticide resistance in mosquitoes is widely accepted [104,105,106] and promoters identified as having neural expression patterns could be used for the functional analysis of SNPs within insecticide-resistant alleles. Given the availability of mosquito genomes and increased transcriptome data, a great number of promoters can be predicted for their ability to drive transgenes in mosquitoes. Additionally, sophisticated genetic tools for expression analysis allow cross-species computational enhancer prediction [13,107,108]. However, these regulatory elements need to be tested before being used to create genetically engineered mosquitoes. A novel artificial-intron-based strategy for mosquito transgenesis supports the co-option of regulatory elements of endogenous loci directly without prior labor-intensive promoter characterization [109] and is a viable approach to satisfy the need of promoters for many infection-relevant tissues.

## 6. Conclusions

Methods to produce transgenic mosquitoes have been available for over 20 years [22,110,111,112]. A number of possible promoters and 5′- and 3′-end DNA sequences to drive the expression of transgenes and effector molecules whose products hinder mosquito population survival or pathogen development have been discovered, yet only a handful of these pre-characterized promoter elements are used routinely for generating transgenic lines intended for population suppression or modification strategies (Table 1). These regulatory sequences can be classified into two groups (Figure 1). The first comprises the ubiquitously expressed promoters, and RNA Pol III promoters (U6) used to generate guide RNA (gRNA)-expressing lines. The second are the tissue-specific promoters, which can drive expression in the fat body, midgut, salivary glands, hemocytes, and testis and/or ovaries, the latter being used to generate Cas9-expressing lines.

Given that the success of transgenic mosquito vector control approaches relies on well-targeted gene expression, the identification and characterization of a diverse set of mosquito promoters and transcriptional enhancers are required for technological progress [13]. An increased knowledge of the expression systems currently used also can help establish dosage-response curves of different types of effectors that may require distinct levels of effectively expressed proteins. Furthermore, the importance of characterizing mosquito regulatory systems goes beyond their use for biotechnology-based approaches, as different sequences acting in each of the life cycle stages of the insect or disease agent can provide valuable insights into mosquito biology and pathogen interaction [128].

Finally, it is important to acknowledge that the application of new genetic engineering technology is challenging because an accepted standard for moving it from the laboratory to the field may not exist or have been tested yet [77]. Pathways for moving gene-drive population suppression and modification mosquitoes to the field are being charted as the work progresses and the science is often ahead of community-based efforts to certify best practices. In response, investigators, scientific advisory groups, and potential stakeholders have offered analyses of challenges and issued guidelines for moving the science forward [129,130,131]. Accepted guiding principles include that the work be conducted in phases in which stringent criteria must be met before moving from one phase to the next. The World Health Organization (WHO) proposed early on a framework for testing genetically engineered mosquitoes and defined four phases: Phase 1 tests are discovery stages physically confined to laboratories and insectaries; Phase 2 moves the strains to development and are carried out in small-scale physically and/or ecologically contained field tests; Phase 3 continues development in a series of open release trials that increase in size, length, and complexity at one or more sites; and Phase 4 moves the technology to a wider application as a malaria control tool in the delivery stage [129]. Specific strains are evaluated and subjected to rigorous ‘go/no go’ criteria in each phase. Later efforts acknowledged the special challenges posed by the gene-drive system [132,133,134]. We encourage all scientists working with these technologies to adopt the principles outlined in these frameworks and make the essential efforts to engage potential stakeholders and end-users [135,136].

## Figures and Tables

**Table 1:** Transgenic mosquitoes exhibiting pathogen refractoriness or lethal/sterile phenotypes.

Mosquito Species	Phenotype	Promoter	References
*An. stephensi*	Pathogen refractoriness	*Cp*	[84,86,87,88,92,113,114,115,116]
		*Aper1*	[83,117]
		*Vg*	[84,85,92,116]
		*aapp*	[98,99,100]
	Lethality/Sterility	*Act-4*	[70]
		*Guy1*	[73]
*An. gambiae*	Pathogen refractoriness	*Cp*	[118,119,120]
		*Vg*	[121,122,123]
	Lethality/Sterility	*β2 tub*	[124]
		*Yob*	[72]
*Ae. aegypti*	Pathogen refractoriness	*Cp*	[86,125,126,127]
		*PUb*	[26,127]
		*30K*	[101]
		*Vg*	[23]
	Lethality/Sterility	*Vg*	[76]
		*Act-4*	[68]
		*Nix*	[74]
*Ae. albopictus*	Lethality/Sterility	*Act-4*	[69]
		*Nix*	[75]

*Cp*, zinc carboxypeptidase A1; *Aper1*, peritrophin; *Vg*, vitellogenin; *aapp*, anopheline antiplatelet protein; *Act-4*, actin-4; *β2 tub*, β2 tubulin; *PUb*, polyubiquitin.
